# Multi-centered T cell repertoire profiling identifies alterations in the immune repertoire of individuals with inflammatory bowel disease across different disease stages

**DOI:** 10.1186/s13073-025-01575-w

**Published:** 2026-01-09

**Authors:** Aya K. H. Mahdy, Hesham ElAbd, Érika Endo Kokubun, Valeriia Kriukova, Mitchell Pesesky, Damon H. May, Christine Olbjørn, Gøri Perminow, May-Bente Bengtson, Petr Ricanek, Svend Andersen, Trond Espen Detlie, Vendel A. Kristensen, Bjørn Moum, Morten H. Vatn, Jørgen Jahnsen, Bernd Bokemeyer, Johannes Roksund Hov, Jonas Halfvarson, Stefan Schreiber, Bryan Howie, Harlan S. Robins, Marte Lie Høivik, Andre Franke, IBSEN-III study group

**Affiliations:** 1 Institute of Clinical Molecular Biology, University of Kiel and University Hospital Schleswig-Holstein, Rosalind-Franklin-Str. 12, 24105 Kiel, Germany; 2 Institute for Digestive Research, Lithuanian University of Health Sciences, Kaunas, Lithuania; 3 Adaptive Biotechnologies, Seattle, Washington USA; 4 Department of Paediatric and Adolescent Medicine, Akershus University Hospital, Oslo, Norway; 5 Department of Pediatrics, Oslo University Hospital, Oslo, Norway; 6 Department of Gastroenterology, Vestfold Hospital Trust, Tonsberg, Norway; 7 Department of Gastroenterology, Lovisenberg Diaconal Hospital, Oslo, Norway; 8 Department of Paediatrics, Vestfold Hospital Trust, Postboks, Tonsberg, Norway; 9 Department of Gastroenterology, Akershus University Hospital, Lørenskog, Norway; 10 Department of Gastroenterology, Oslo University Hospital, Oslo, Norway; 11 Institute of Clinical Medicine, Faculty of Medicine, University of Oslo, Oslo, Norway; 12 Østfold Hospital, Kalnes, Norway; 13 Interdisciplinary Crohn Colitis Centre, Minden, Germany; 14 Norwegian PSC Research Center and Section of Gastroenterology, Department of Transplantation Medicine, Division of Surgery and Specialized Medicine, Oslo University Hospital, Oslo, Norway; 15 Research Institute of Internal Medicine, Division of Surgery and Specialized Medicine, Faculty of Medicine, Institute of Clinical Medicine, University of Oslo, Oslo, Norway; 16 Department of Gastroenterology, Faculty of Medicine and Health, Örebro University, Örebro, Sweden

## Abstract

**Background:**

Inflammatory bowel disease (IBD) is an incurable immune-mediated inflammatory disease, affecting the gut with a high rate of primary- and secondary- loss-of-response to therapy. By investigating the T cell receptor repertoire of individuals with IBD, novel therapeutic and preventive strategies can be identified, and a better understanding of IBD can be obtained.

**Methods:**

To identify and validate T cell clonotypes implicated in the pathogenesis of IBD, we profiled the T cell receptor alpha (TRA) repertoire of three cohorts containing treatment-naive, treated individuals, and individuals living with the disease for >20 years, resulting in an exhaustive dataset containing the TRA repertoire of 1,732 individuals.

**Results:**

Using the generated datasets, we were able to replicate previous findings describing the expansion of Crohn’s-associated invariant T (CAIT) cells in individuals with Crohn’s disease (CD) in the three cohorts. Using a hypothesis-free statistical testing framework, we identified clonotypes that were associated with the disease at its different stages, *e.g.,* at the time of diagnosis and decades post-diagnosis. By conducting a meta-analysis across the three cohorts, we were able to identify a set of clonotypes that were associated with the disease regardless of its stage. We validated our findings in a previously published independent test dataset from a German cohort, showing the robustness of the identified clonotypes.

**Conclusions:**

The identified clonotypes are novel therapeutic targets to treat IBD, for example, through targeted depletion. By identifying antigens recognized by these T cells, a better understanding of the etiopathology of IBD, particularly CD, can be obtained.

**Supplementary Information:**

The online version contains supplementary material available at 10.1186/s13073-025-01575-w.

## Background

Inflammatory bowel disease (IBD) is associated with a significant reduction in the quality-of-life and increased morbidity [1, 2]. Although different therapies have been developed to treat IBD, such as anti-TNF and anti-integrins, they fail to induce a response in all patients, *i.e.,* primary non-responders [3, 4]. Furthermore, loss-of-response is commonly observed, as patients develop antibodies against these medications [3]. Thus, there is an urgent need to develop novel therapies that induce long-lasting remission in a large fraction of patients. Cellular immunotherapies have shown promising results in treating immune-mediated inflammatory diseases (IMIDs), for example, B cell depletion via chimeric antigen receptor (CAR) T cells has been shown to be a robust tool to treat lupus nephritis [5]. Similarly, chimeric autoantibody receptor (CAAR) T cells have been successfully used to treat pemphigus vulgaris, offering a more specific approach by depleting only B cell clones involved in the disease [6]. A prerequisite for developing CAAR T cells is identifying antigens targeted by disease-relevant B cell clones. However, in IBD, the antigen(s) driving the disease remain to be elucidated. A novel approach that targets disease-associated T cell clones, regardless of their antigenic specificity, was recently reported by Britanova *et al.* [7] for ankylosing spondylitis. This approach relies on using an antibody to deplete all disease-implicated clonotypes and has illustrated promising results in early-stage trials [7]. Thus, disease-associated T cell clones are a novel therapeutic target for treating IMIDs such as IBD. In addition, they provide a powerful framework to identify antigenic exposures implicated in diseases, for example, by profiling the TCR repertoire of 504 individuals with primary sclerosing cholangitis (PSC) and 904 controls, we identified multiple clonotypes that were implicated in PSC [8]. Using several approaches, we showed that a subset of these PSC-associated clonotypes targeted Epstein-Barr virus, illustrating the utility of large-scale repertoire profiling in understanding the etiopathology of IMIDs [8, 9].

However, identifying T cell clonotypes that are involved in IBD is not a trivial task for multiple reasons, such as heterogeneities in the clinical presentation, differences in affected tissues, and a complicated genetic architecture, particularly within the human leukocyte antigen (HLA) loci. Multiple HLA alleles have been associated with IBD, for example, HLA-DRB1*01:03 [10], or with one of IBD’s subsets, *i.e.,* Crohn’s disease (CD) and ulcerative colitis (UC), such as HLA-DRB1*07:01, which is strongly associated with CD [11] and HLA-DRB1*15:01 with UC [12]. Furthermore, each patient experiences a different journey that is characterized by different medication use as well as different surgeries. By profiling the T cell repertoire of individuals with IBD, multiple alterations were identified, such as altered responses toward yeast antigens [13] and gut microbiota [14], as well as an expansion of a subset of type II invariant natural killer T (NKT) cells particularly in individuals with CD [15]. These cells are termed CAIT cells and are defined using their semi-invariant TCR alpha chain with a *TRAV12-1* and *TRAJ6* gene usage and the following CDR3 amino acid motif: CVV**A*GGSYIPTF. Although CAIT cells were identified as a group of expanded clonotypes using bulk TCR repertoire analyses, their phenotype was decoded using single-cell transcriptomic analyses, with a gene expression that resembles that of unconventional T cells, particularly NKT cells. Whereas the exact antigen(s) driving the expansion of CAIT cells in individuals with CD have not been identified yet, we previously showed that CAIT cells can recognize small molecules such as PPBF and CIPPBF presented by CD1d molecules [16]. Other alterations have been identified in individuals with IBD, such as the presence of multiple expanded clonotypes in the colonic mucosa of individuals with CD [17, 18]. Advances in bulk T cell repertoire sequencing (TCR-Seq) [19] and statistical analyses have enabled public clonotypes associated with a particular antigenic exposure, such as cytomegalovirus [20], SARS-CoV-2 [21], Lyme disease [22], and other IMIDs such as PSC [8], to be identified.

Most of these studies have focused on the more diverse beta chain of the T cell receptor, *i.e.,* the TRB repertoire, leaving the less diverse alpha chain repertoire, * i.e. *the TRA repertoire, mostly unexplored. From a statistical perspective, studying the TRA repertoire is more promising as a smaller sample size might be needed to identify alterations driving the disease. Furthermore, most unconventional T cells, such as mucosal-associated invariant T (MAIT) cells, are characterized by a semi-invariant TRA chain; thus, the expansion of these cells can be easily quantified from the TRA repertoire. Hence, we aimed to utilize TCR-Seq to identify and validate public clonotypes associated with different subsets of IBD at different stages and different disease trajectories.

## Methods

### Cohort description

We profiled the TRA repertoire of three distinct cohorts from Germany and Norway.

**Fig. 1:**
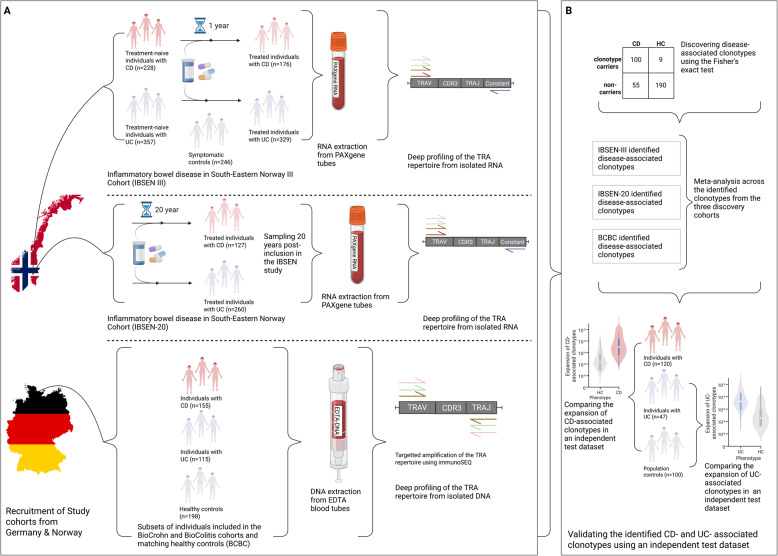
The included cohorts and the analytical pipeline used in the current study. **A** the pipeline for profiling the TRA repertoire of the three discovery cohorts, namely, IBSEN-III, IBSEN-20, and the BCBC cohort. **B** The analytical framework used for identifying disease-associated clonotypes from the three discovery cohorts, as well as among the three cohorts using a meta-analysis approach. Lastly, the identified clonotype-sets were validated using a previously published test dataset [15]. The figure was created in BioRender. ElAbd, H. (2025) https://BioRender.com/rc6hedu
II. **The IBSEN-20 cohort**
  The IBSEN-20 cohort (Additional file 2: Table S2)- contains patients included in the first IBSEN study, Norway [24], 20 years post-diagnosis (Fig. 1A). The cohort contains 127 individuals with CD and 260 individuals with UC (Fig. 1A).
III. **The BCBC cohort**
  The BioCrohn and BioColitis cohorts- (BCBC cohort; Additional file 2: Table S3) contains 155 individuals with CD and 115 individuals with UC from Germany in addition to 198 population controls also from Germany.

I. **The IBSEN-III cohort**
  The IBSEN-III cohort (Additional file 2: Table S1) contains treatment-naive and treated individuals with IBD from Norway, in addition to individuals with symptoms of IBD but without any radiological or endoscopic findings, *i.e.,* symptomatic controls (Fig. 1A) [23]. The cohort contained 228 treatment-naive individuals with CD and 357 with UC, in addition to 246 symptomatic controls. The cohort also contained individuals one year after diagnosis and treatment, specifically, 176 individuals with CD and 329 individuals with UC. The cohort also contained paired measurements at baseline and one-year after treatment for 160 individuals with CD and 299 individuals with UC.

### Study design

From both Norwegian cohorts, *i.e.* the IBSEN-III and the IBSEN-20, PAXgene Blood RNA tubes were collected and used for RNA extraction. Subsequently, the RNA was used to profile the TRA repertoire (Fig. 1A). For the German BCBC cohort, DNA was extracted from EDTA blood tubes and then utilized to profile the TRA repertoire (Fig. 1A). After that, we used a hypothesis-free statistical framework described by Emerson and colleagues [20] to identify sets of clonotypes that are associated either with CD or UC. Subsequently, we performed a meta-analysis on the identified clonotypes from each cohort to identify a robust set of disease-associated clonotypes. Lastly, we used a previously published test dataset [15] to validate the identified CD- and UC- associated clonotypes (Fig. 1B).

### TCR profiling using DNA

The TRA repertoire of the BCBC cohort and matched population controls was profiled using DNA extracted from peripheral blood. Subsequently, up to 18 µg of DNA per sample were used for profiling the TRA repertoire using the immunoSEQ assay (Adaptive Biotechnologies).

### TCR profiling using RNA

The TRA repertoires of the IBSEN-III and the IBSEN-20 cohorts were profiled using RNA extracted from PAXgene Blood RNA tubes collected from peripheral blood. From the IBSEN-III cohort, up to 300 ng of RNA were used, while for the IBSEN-20, up to 200 ng of RNA were used. Subsequently, next-generation sequencing (NGS) libraries of the PCR-amplified TRA repertoire were generated using MiLaboratories’ commercially available kits according to the manufacturer’s instructions. After indexing the samples using Illumina dual indices, samples were pooled together and sequenced using 150 bp paired-end sequencing on the NovaSeq 6000. After demultiplexing the generated sequencing reads , *i.e.,* FASTQ files, the sequencing reads of each samples were processed using MiXCR [25] (v4.6) to identify and quantify the expansion of the different TRA clonotypes present in each sample.

### Processing the identified clonotypes and generated repertoires

After identifying TRA clonotypes using either the immunoSEQ assay or MiLaboratories kits, we processed the repertoires by removing non-productive clonotypes, *i.e.,* clonotypes containing a frameshift or a stop codon, and hence they do not encode for a functional TRA chain. Subsequently, we grouped different VJ recombination encodings for the same TRA chain at the protein level into a single clonotype and summed their expansion. That is, each clonotype included in the analysis represented a unique VJ recombination with a unique CDR3 amino acid sequence in a sample. Lastly, we removed samples with fewer than 1,000 productive clonotypes.

### Identifying CD- and UC- associated clonotypes

To identify TRA clonotypes that are associated with CD or UC, we utilized the framework described by Emerson *et al.* [20], focusing on public clonotypes, *i.e.,* clonotypes present in more than one individual. After identifying public clonotypes, we compared their frequency in cases, *e.g.,* CD or UC, and controls using a one-sided Fisher’s exact test. Subsequently, we used a cutoff of 1x10^−3^ to identify associated clonotypes. For both the IBSEN-III and the BCBC cohorts, we compared the repertoire of CD and UC individuals to symptomatic controls or to healthy controls, respectively, to identify CD- and UC- associated clonotypes. Meanwhile, for the IBSEN-20, neither healthy nor symptomatic controls were available, and we compared the repertoire of CD to that of UC and vice versa to identify clonotypes associated with either CD or UC.

### Seeded clustering of TRA-associated clonotypes

To extend the set of disease-associated clonotypes to rarer clonotypes that our study did not have the statistical power to identify, we performed seeded clustering as described previously [8]. Briefly, this clustering step is composed of three steps:

1. Identifying disease-associated clonotypes using the Fisher’s exact test as defined above (identifying CD- and UC-associated clonotypes). These clonotypes represent the seeds, which are the base for extending the clonotype search on.
2. Extended search: after identifying the seeds, that is CD- or UC- associated clonotypes, for each seed, we searched all repertoires for clonotypes that have the same V and J genes as that of the seed and a CDR3 amino acid sequence that is at max 1-Levenshtein distance from the seed. The collection of a seed and its similar sequences is referred to as an unpurified meta-clonotype.
3. Seed purification, to purify and define the final set of meta-clonotypes, we iterated over each member of the unpurified meta-clonotypes, where we compared the association *P*-value of the seed and a given member of the unpurified meta-clonotypes to that of the seed using a one-sided Fisher’s exact test. If the *P*-value of the member and the seed was larger than the *P*-value of the seed alone, then this member is excluded from the unpurified meta-clonotype. Otherwise, it is kept. The set of clonotypes that survive the purification step, and their seed, are referred to as the purified meta-clonotype.

### Performing meta-analysis across the different cohorts

To perform a meta-analysis across the three cohorts described in the study, namely, the IBSEN-III, the IBSEN-20, and the BCBC cohort, we utilized Fisher’s combined *P*-value approach. After identifying the clonotypes associated with either CD or UC from each cohort independently, *i.e.,* seeds, we focused on the clonotypes that are present in the three cohorts. Subsequently calculated an association *P*-value for each cohort using the one-sided Fisher’s exact test. Thus, we ended up with three *P*-values for each clonotype that was detected in the three cohorts. These *P*-values were combined using the Fisher’s approach to generate a single *P*-value [26, 27]. Lastly, we utilized the Benjamini-Hochberg correction method to correct for multiple testing and adjust the *P*-value. TRA clonotypes with an adjusted *P*-value <0.05 were identified as CD- or UC- associated clonotypes identified from the meta-analysis.

### Graph analysis of the identified clonotypes

To perform a network analysis of the identified CD- and UC- associated clonotypes, we used a graph-based approach in which clonotypes were represented as nodes and edges reflected similarity between these nodes. Two nodes, *i.e.,* TRA clonotypes, were connected by an edge if they shared the same V and J genes and had a Hamming distance of one between their CDR3 amino acid sequences. Visualization of the resulting graph was performed using Cytoscape (v3.10.3) [28].

### Statistical analyses

We utilized different statistical analyses. Specifically, for identifying disease-associated clonotypes, we used the one-sided Fisher’s exact test (FET) where the significance level was set to 1x10^−3^. The FET was also used in the seeded clustering analysis to identify clusters of highly similar sequences that are implicated in the disease. To compare different groups, we used the two-sided Mann-Whitney U test with a default significance level of 0.05. For paired group comparisons, we utilized the paired Wilcoxon test with the commonly used significance level of 0.05. For the meta-analysis, we used the Fisher’s combined P-value method [26, 29] with the Benjamini-Hochberg correction for multiple testing. We used the default implementation of these methods within commonly used software in the Python (v3.10) ecosystem, namely, pandas, NumPy, SciPy and the scikit-learn library.

## Results

### CAIT cells are expanded across different disease stages and are not induced by treatment

**Fig. 2:**
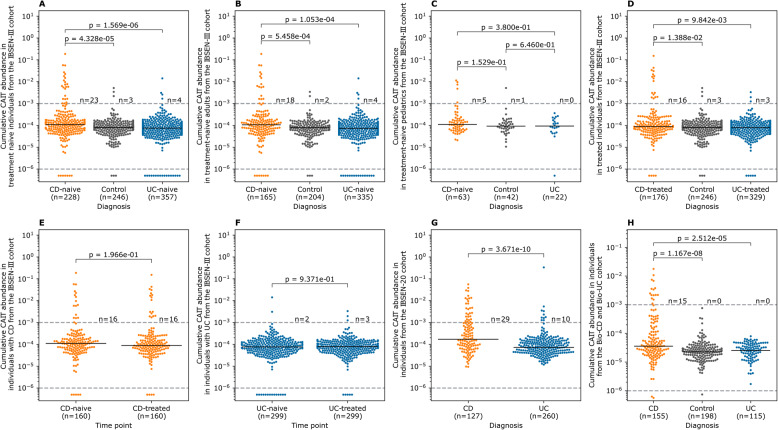
The expansion of CAIT cells in different cohorts and phenotypic groups. **A** expansion of CAIT cells in treatment-naive individuals with CD or UC, as well as symptomatic controls from the IBSEN-III cohort. **B** and **C** expansion of CAIT cells in the same phenotypic groups shown in (**A**) but separated by age, with (**B**) showing the expansion in adults (age>18 years old) and (**C**) showing the expansion in pediatric samples (age<18 years old). **D** expansion of CAIT cells in treated individuals with either CD or UC, as well as symptomatic controls. **E** and **F** expansion of CAIT cells in a subset of individuals from the IBSEN-III cohort with paired measurements of their T cell repertoires before and after treatment. **G** expansion of CAIT cells in individuals with CD or UC from the IBSEN-20 cohort. **H** expansion of CAIT cells in individuals with CD relative to UC and healthy controls from the BCBC cohort. In panels (**E**) and (**F**), the expansion of CAIT cells before and after treatment was compared using the paired Wilcoxon test, while in all other panels, the two-sided Mann-Whitney U test was used to compare the expansion of CAIT cells among the different groups, further, an α-level of 0.05 was used to define the significance level for all statistical tests

We first aimed to study the expansion patterns of Crohn’s-associated invariant T (CAIT) cells in individuals with IBD as well as in healthy controls. Given that CAIT cells can recognize small molecules that resemble drugs and/or bacterial metabolites [16], we compared the expansion of CAIT cells in treatment-naive individuals with IBD. Using the TRA repertoire of the IBSEN-III cohort, we observed that CAIT cells were expanded in treatment-naive individuals with CD relative to treatment-naive individuals with UC and symptomatic controls (Fig. 2A). Because the IBSEN-III cohort contains both treatment-naive adults and children with IBD, these groups were studied separately. CAIT cells were significantly expanded in adults with CD relative to UC or symptomatic controls (Fig. 2B) but not in pediatric cases (Fig. 2C). This might be a consequence of differences in the sample size or a true biological difference in the pathogenesis of adult and pediatric forms of IBD.

To quantify the effect of treatment on the expansion of CAIT cells, we compared their expansion in treated individuals, which recapitulated the findings observed in treatment-naive individuals (Fig. 2D). Indeed, by focusing on only individuals with paired measurements, *i.e.,* before treatment and one year after treatment, we observed that CAIT cells had a comparable level of expansion in treated and treatment-naive individuals with CD (Fig. 2E) or UC (Fig. 2F). This suggests that treatment had a minor impact on the expansion of CAIT cells. Using the TRA repertoire of the IBSEN-20 cohort, we observed a significant expansion of CAIT cells in individuals with CD relative to individuals with UC (Fig. 2G). This was also replicated in the German BCBC cohort, which showed a significant expansion of CAIT cells in individuals with CD relative to healthy controls and individuals with UC (Fig. 2H). Thus, by profiling the T cell repertoire of three different cohorts from different geographical locations and using different TCR-Seq methodologies, we observed a significant expansion (P_meta CD vs. controls_ = 1.4x10^−11^; P_meta CD vs. UC_ = 1.57 x 10^−17^) of CAIT cells in individuals with CD relative to UC, corroborating previous findings [15].

### The expansion of CAIT cells is higher in ASCA^+^ individuals with CD and in individuals with ileal involvement and penetrating disease behavior

**Fig. 3:**
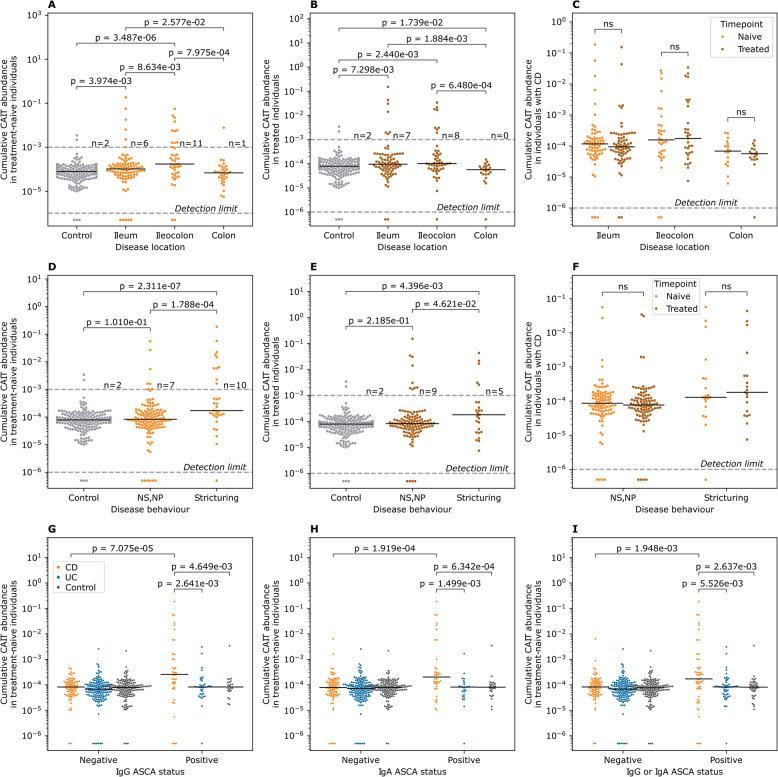
Subphenotypes and serological markers associated with high levels of CAIT expansion in adult individuals from the IBSEN-III cohort. **A** and **B** expansion of CAIT cells in treatment-naive (**A**) and treated individuals (**B**) with different forms of CD and symptomatic controls. **C** expansion of CAIT cells in individuals with CD before and after treatment using paired measurements from the same individuals. **D** and **E** show the expansion of CAIT cells in symptomatic controls and individuals with CD with different disease behaviors. **F** minor impact of treatment on the expansion of CAIT cells in individuals with different disease behaviors. **G** expansion of CAIT cells in ASCA^+^ individuals as measured via IgG, while (**H**) depicts the same relationship but according to IgA-based measurements. **I** relationship between ASCA-positivity and the expansion of CAIT cells, by defining positivity as either IgG or IgA positive

After validating the robustness of the CAIT signal across different cohorts, we aimed to investigate subphenotypes associated with a higher CAIT expansion, focusing on adult individuals from the IBSEN-III cohort. Across treatment-naive and treated individuals, CAIT cells were significantly expanded in individuals with ileal involvement, *i.e.,* ileal and ileocolonic CD (Fig. 3A & B). This location-specific expansion was not affected by medications, as the expansion was comparable in the same individuals before and after treatment (Fig. 3C). Disease behavior also correlated with CAIT expansion where it was higher in individuals with stricturing disease relative to individuals without a stricturing or a penetrating disease either at the treatment-naive or the treated stage (Fig. 3D & E). Furthermore, the expansion of CAIT cells was comparable in individuals with CD but without a stricturing or a penetrating disease, and controls. This indicates that the severity and the anatomical location of the disease are the major factors governing the expansion of CAIT cells and that treatment had a minor impact on the expansion of these cells (Fig. 3F). In addition, ASCA status strongly correlated with the expansion of CAIT cells only in individuals with CD, which was evidenced at the IgG (Fig. 3G) and IgA (Fig. 3H) levels as well as when considering either of them (Fig. 3I).

### Mucosal-associated invariant T (MAIT) cells are significantly reduced in the blood of individuals with IBD relative to symptomatic controls

A reduction in the expansion of MAIT cells has been previously reported in individuals with IBD [15, 30]. To investigate if this effect is related to medication intake or the underlying disease, we compared the expansion of MAIT cells, defined as TRAV1-2+TRAJ33^+^ clonotypes, in treatment-naive and treated individuals from the IBSEN-III cohort. The expansion of MAIT cells was reduced in individuals with CD or UC but was comparable between treatment-naive and treated individuals (Additional file 1: Figure S1A). While the expansion of MAIT cells was comparable between males and females with UC or in controls, it was lower in males with CD relative to females with CD (Additional file 1: Figure S1B). Across the different diseases, the abundance of MAIT cells negatively correlated with age, indicating that age, biological sex, and disease status can all influence the expansion of MAIT cells, and that treatment has a minor impact on the expansion of these cells.

### Hypothesis-free statistical analyses confirm previous findings and identify novel clonotypes that are associated with either CD or UC

Next, we aimed to identify other clonotypes associated with either CD or UC using a hypothesis-free statistical association framework (Methods). This analysis revealed 38, 72, and 13 clonotypes that were associated with CD and 35, 70, and 1 clonotypes that were associated with UC in the IBSEN-III cohort, the IBSEN-20, or the BCBC cohort, respectively. A common theme among the different sets was the detection of multiple CAIT-like clonotypes, *i.e.,* TRA chains that followed the same CAIT motif in terms of V and J gene usage and CDR3 amino acid sequence. Specifically, 2 out of the 38 CD-associated clonotypes identified from the IBSEN-III cohort, 7 out of the 72 CD-associated clonotypes identified from the IBSEN-20 cohort, and 2 out of the 13 CD-associated clonotypes identified from the BCBC cohort were CAIT clonotypes, indicating the robust association of CAIT cells with CD. However, these sets of CD-associated clonotypes did not show a robust overlap with each other (Additional file 1: Figure S2A). A similar pattern was seen among the sets of UC-associated clonotypes (Additional file 1: Figure S2B). This could have multiple explanations, such as the stage of the disease, where different clonotypes are involved in the disease at different stages, *e.g.,* the early stage observed in the IBSEN-III cohort relative to the late stage observed in the IBSEN-20 cohort. Alternatively, this can be attributed to differences in the sample size among the different cohorts and hence differences in the statistical power, or a combination of these two factors.

To extend our analysis to rarer disease-associated clonotypes that we were not able to identify statistically because of the relatively small sample size of each cohort, we performed seeded clustering (Methods). This enabled us to identify clonotypes with a similar sequence and directionality but a lower magnitude of expansion than the clonotypes identified from the initial analysis. This extended the number of CD-associated clonotypes to 111, 230, and 240 clonotypes arranged into 38, 72, and 13 meta-clonotypes derived from the IBSEN-III, the IBSEN-20, and the BCBC cohort, respectively. Similarly, this extended the number of UC-associated clonotypes to 122, 340, and 3 clonotypes, arranged into 35, 70, and 1 meta-clonotypes, respectively. Still, limited overlap was observed between the CD-associated clonotype sets (Additional file 1: Figure S3A) as well as the UC-associated clonotype sets (Additional file 1: Figure S3B).

**Fig. 4:**
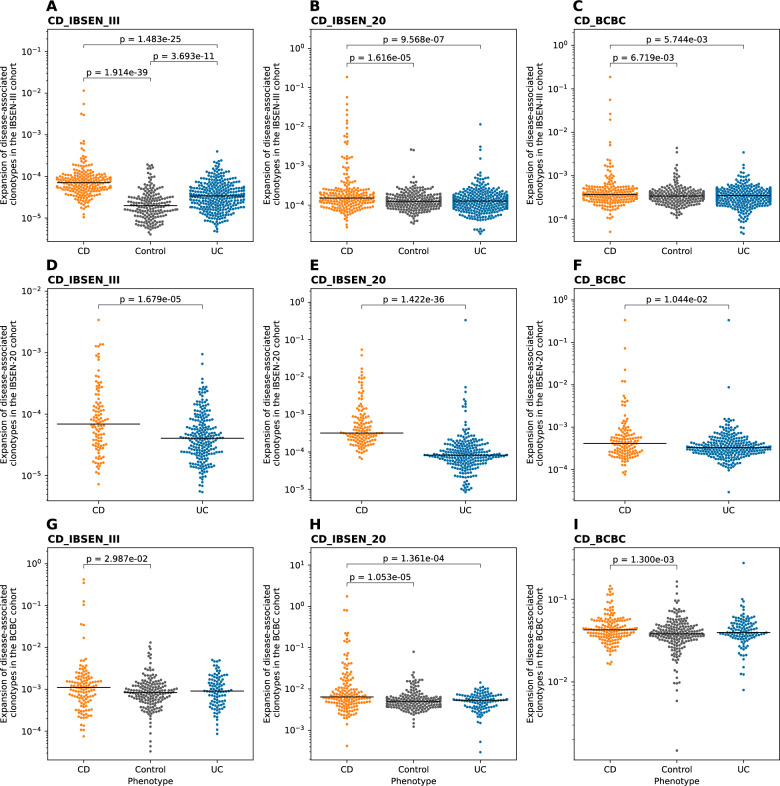
Expansion of the different CD-associated clonotypes sets in the three study cohorts, namely, IBSEN-III, IBSEN-20, and BCBC. **A**, **B** and **C** expansion of the three CD-associated clonotype sets identified by analyzing the treatment-naive IBSEN-III cohort (CD_IBSEN_III), the IBSEN-20 cohort (CD_IBSEN_20), and the BCBC cohort (CD_BCBC cohort) in the treatment-naive IBSEN-III dataset. **D**, **E**, and **F** expansion of these three CD-associated sets in the IBSEN-20 dataset, while **G**, **H**, and **I** expansion of these CD-associated clonotypes in the BCBC cohort

Before we investigated these clonotype sets further, we aimed to validate their expansion in their respective phenotypes, *e.g.,* CD-associated clonotypes in individuals with CD, among the three discovery cohorts. The expansion of CD-associated meta-clonotypes identified from the treatment-naive samples from the IBSEN-III cohort (CD_IBSEN_III) was significantly higher in individuals with CD relative to symptomatic controls and individuals with UC from the treatment-naive IBSEN-III cohort (Fig. 4A). Within the same cohort, the expansion of CD-associated meta-clonotypes identified from the IBSEN-20 cohort (CD_IBSEN_20) was significantly higher in individuals with CD relative to the other two groups (Fig. 4B). Additionally, the expansion of CD-associated meta-clonotypes identified from the BCBC cohort (CD_BCBC) was higher in individuals with CD relative to individuals with UC and controls (Fig. 4C). These findings indicate that the expansion of three sets of CD-associated meta-clonotypes, *i.e.* CD_IBSEN_III, CD_IBSEN_20 and CD_BCBC was significantly higher in individuals with CD included in the IBSEN-III cohort relative to individuals with UC and symptomatic controls. The same pattern was observed when comparing the expansion of these meta-clonotypes sets in the other two discovery cohorts, namely, the IBSEN-20 cohort (Fig. 4D, E, and F) and the BCBC cohort (Fig. 4 G, H, and I). This indicates that these sets of CD-associated meta-clonotypes are robustly associated with CD and are an immunological fingerprint for CD.

There were notable discrepancies among the identified UC-associated meta-clonotype sets. Using the BCBC cohort, we were able to identify only one meta-clonotype as associated with UC, potentially due to the small sample size (*n*=115 individuals). Hence, we focused our analysis on two UC-associated meta-clonotype sets: the first set was derived from the treatment-naive IBSEN-III cohort (UC_IBSEN_III) and the second from the IBSEN-20 (UC_IBSEN_20). Within the IBSEN-III dataset, the expansion of the UC_IBSEN_III meta-clonotype set was significantly higher in individuals with UC relative to individuals with CD and symptomatic controls (Additional file 1: Figure S4A). However, the expansion of the UC_IBSEN_20 set was significantly higher in symptomatic controls than in individuals with CD or UC (Additional file 1: Figure S4B). Similarly, the expansion of the UC_IBSEN_III set was comparable in individuals with UC and CD included in the IBSEN-20 cohort (Additional file 1: Figure S4C), but the expansion of the UC_IBSEN_20 set was higher in individuals with UC relative to individuals with CD from this cohort (Additional file 1: Figure S4D). Lastly, within the BCBC cohort, the UC_IBSEN_III meta-clonotype was predominantly expressed in individuals with UC (Additional file 1: Figure S4E), while the UC_IBSEN_20 showed the highest expansion in healthy controls relative to individuals with either CD or UC (Additional file 1: Figure S4F). Thus, within the two sets, the UC_IBSEN_III was significantly expanded in individuals with UC in its discovery cohort (IBSEN-III) and an independent validation cohort (BCBC). On the contrary, the UC_IBSEN_20 set was only expanded in individuals with UC in its discovery cohort (*i.e.*, IBSEN-20) and neither of the other two validation cohorts.

This might be attributed to two interwoven reasons: first, the type of statistical comparisons used to identify UC-associated clonotypes between the IBSEN-III and the IBSEN-20. In the former, *i.e.,* the IBSEN-III cohort, UC-associated clonotypes were identified by comparing the repertoire of individuals with UC to that of controls; meanwhile, in the latter, *i.e.,* IBSEN-20, the clonotypes were identified by comparing the repertoire of individuals with UC to that of CD. Hence, the clonotypes identified from the IBSEN-20 cohort might represent a non-CD signal instead of a set of clonotypes that are associated with UC. On top of this, individuals in the IBSEN-20 cohort are generally older and have a more advanced disease course and a more complicated treatment trajectory, which might weaken or bias the disease-signal in individuals with UC.

### Meta-analysis enables the identification of a robust set of CD- and UC- associated clonotypes

After identifying and validating the expansion of the different CD-associated meta-clonotypes and a subset of the UC-associated meta-clonotypes, we aimed to integrate and unify these sets. Thus, we performed a meta-analysis across the three sets (Methods). Specifically, for each clonotype belonging to the union of the CD- or UC- associated clonotypes, we calculated an association *P*-value using the Fisher’s exact test in each of the three discovery cohorts. Subsequently, we combined the calculated *P*-values using the Fisher's combined probability approach and, lastly, corrected for multiple testing using the Benjamini-Hochberg procedure (Methods). Focusing on the three CD-associated clonotype sets and the approach outlined above, we identified 25 clonotypes that were associated with CD (adjusted *P*-value <0.05; Additional file 2: Table S4). Seven out of these 25 clonotypes (~28%) were CAIT clonotypes, corroborating previous findings about the relevance of these cells to the pathology of CD. We used the same meta-analysis-based framework to identify clonotypes that are associated with UC, focusing on the two UC-associated clonotype sets identified from the IBSEN-20 cohort and the IBSEN-III cohort, which enabled us to identify 76 public TRA clonotypes that were associated with UC (Additional file 2: Table S5). We also repeated the same process but excluded the IBSEN-20 cohort, which enabled the identification of 27 clonotypes that were associated with UC.

**Fig. 5:**
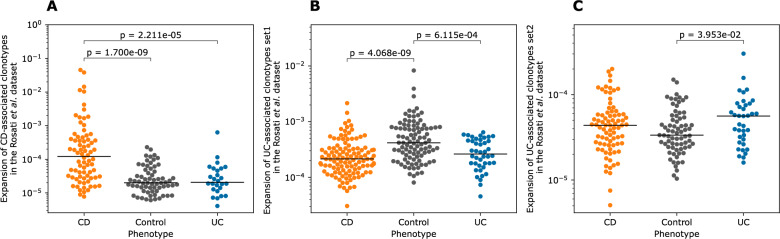
The expansion of the identified CD- and UC- associated clonotype sets using an independent test dataset [15]. **A **expansion of the CD-associated clonotypes in individuals with CD relative to individuals with UC and healthy controls. **B **and **C** expansion of the two sets of UC-associated clonotypes, *i.e.*, set 1 and set 2 described above, respectively, in individuals with either CD or UC as well as healthy controls

Before we investigated these new clonotype sets, we aimed to validate their expansion using an independent test dataset. To this end, we used a previously published dataset by Rosati *et al.* [15], which contained the TRA repertoire of 120 individuals with CD, 47 with UC, and 100 population controls. The expansion of the CD-associated clonotype set identified via the meta-analysis described above (*n*=25) was significantly higher in individuals with CD relative to healthy controls and individuals with UC (Fig. 5A). This confirmed that these clonotypes capture a reproducible fraction of CD's immune signature. We had two UC-associated clonotype sets, which were derived by including the IBSEN-20 in the analysis (*n*=76; set-1) or by excluding the IBSEN-20 cohort from the analysis (*n*=27; set-2). The expansion of set-1 was higher in healthy controls relative to individuals with CD and individuals with UC (Fig. 5B). The expansion of set-2 was significantly higher in individuals with UC relative to healthy controls (Fig. 5C) despite containing a smaller number of clonotypes (*n*=27). These findings indicate that the identified clonotypes are specific to CD and UC, respectively.

### CD- and UC- associated clonotypes belong to multiple distinct clusters

**Fig. 6:**
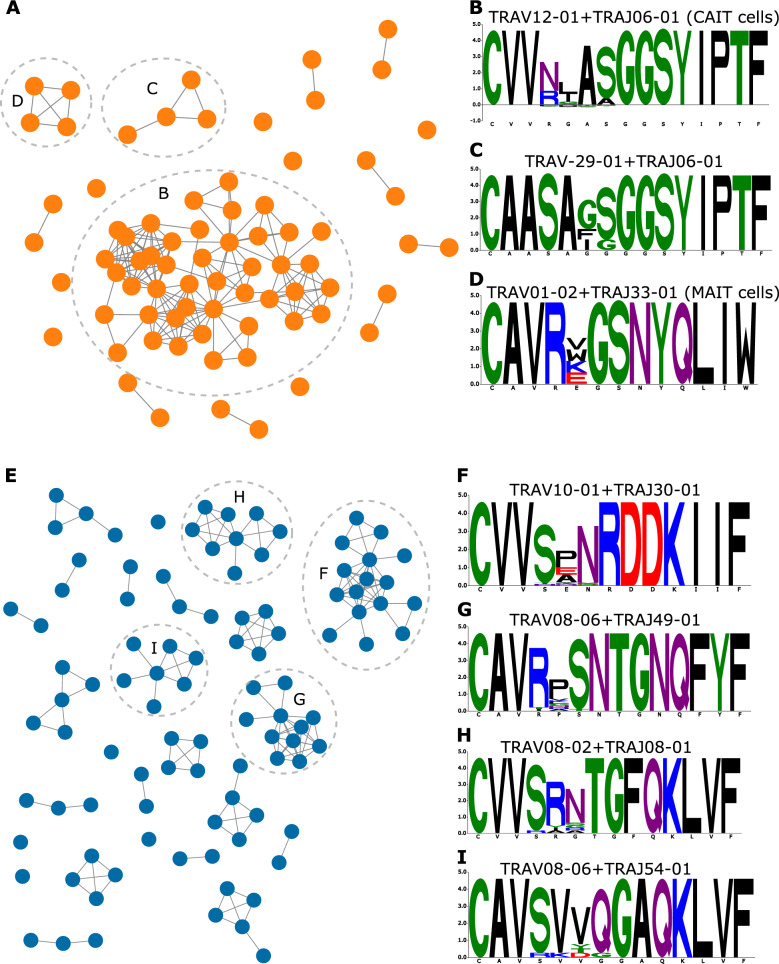
Network analysis of CD- and UC- associated clonotypes (set 2) identified from the meta-analysis conducted across the IBSEN-III, the IBSEN-20, and the BCBC cohort. **A** graph-based representation of CD-associated clonotypes where nodes represent clonotypes, while edges represent similarity among these clonotypes; specifically, two nodes are connected if they have the same V and J genes and their CDR3 is different by only one Hamming distance. **B**-**D**, motif representation of the CDR3 amino acid sequence of the three lergest CD-associated clonotypes depicted in (**A**). **E** network representation of the UC-associated meta-clonotypes (set 2) identified from the cross-cohorts meta-analysis. **F**-**I** CDR3-amino acid motif for the four largest UC-associated clusters

After validating the identified CD- and UC- associated clonotypes using a meta-analysis as well as using an independent test dataset, we aimed to understand the relationship among these clonotypes. Given that we performed our meta-analysis on the initial hits prior to seeded clustering, we augmented these sets with their corresponding meta-clonotypes and then performed a graph-based analysis (Methods). Starting with CD-associated meta-clonotypes, we observed multiple distinct clusters (Fig. 6A), the largest of them belonged to CAIT cells as they share the same V and J gene combination and CDR3 amino acid motif (Fig. 6B). The second biggest cluster has a *TRAV29-01* and *TRAJ06-01* based combination and the following CDR3 amino acid motif (CAASA**GGSYIPTF) (Fig. 6C). Lastly, the third biggest cluster showed a MAIT-like VJ recombination that is derived from *TRAV01-02* and *TRAJ33-01* as well as a conserved CDR3 amino acid motif that only varied in a single amino acid position (Fig. 6D).

There were also multiple distinct clusters identified from the UC-associated meta-clonotypes (Fig. 6E). The biggest of these clusters were derived from a combination between *TRAV10-01* and *TRAJ30-01* with a CDR3-motif that predominantly differed in only one amino acid position (Fig. 6F). There were also multiple clusters derived from the TRAV08 family, for example, the second biggest cluster was derived from a combination between *TRAV08-06* and *TRAJ49-01* and CDR3 amino acid motif that differed in one amino acid position (Fig. 6G). The third biggest cluster was a composite of a VJ recombination between the *TRAV08-02* and *TRAJ08-01* segments and a CDR3-amino acid motif that differed on only two amino acid positions (Fig. 6H). The last cluster with more than seven clonotypes was derived from a *TRAV08-06* and *TRAJ54-01* based recombination; the members of this cluster showed a conserved CDR3 motif with a potential variation in one to two positions (Fig. 6I).

After identifying these clusters, which indicate a focused immune response toward multiple distinct antigens, we aimed to investigate the antigenic specificity of these clonotypes. Using a yeast-specific TCR sequences dataset [13], we detected multiple overlaps with the set of CD-associated clonotypes, particularly with CAIT cells (*n*=14 clonotypes). There was no overlap with UC-associated clonotypes, which is consistent with the fundamental role of anti-fungal responses in CD but not in UC. Through the utilization of T cell receptor-antigen interactions databases (TRAIT) [31], which is a recently published dataset of TCR sequences with their antigenic specificity, we were able to identify the antigenic target of one CD-associated clonotype that was restricted by a SARS-CoV-2 peptide presented by the HLA-A*02 protein. Several factors could explain this, such as cross-reactivity between the antigen(s) recognized by these TRA clonotypes and SARS-CoV-2, and noise in the public annotation database. Similarly, we could not infer the antigenic specificity of any of the UC-associated clonotypes.

## Discussion

Several studies previously aimed at identifying antigens and risk factors potentially causing IBD [13, 14, 32]. Although some risk factors have been identified, *e.g.,* antibiotic intake [33], and infectious mononucleosis [34], the etiology of the disease remains far from understood. While TCR-Seq does not enable the direct identification of these antigens, it can pinpoint their trace in the adaptive immune system by identifying clonotypes recognizing these antigens [19, 20]. Across the three cohorts included in the study, we observed a significantly higher expansion of CAIT cells in individuals with CD, particularly in individuals with ileal involvement. This expansion was consistent across different disease stages as well as in treatment-naive and treated individuals, suggesting that the expansion of CAIT cells is an integral component of the disease. From a therapeutic perspective, CAIT cells are a promising target because they are restricted by the CD1d molecule, which is mostly monomorphic, implying that a CAIT-targeting therapy can be utilized in a larger cohort of affected individuals. This is in contrast to conventional T cells which are restricted by a specific allele, implying that any medication affecting any conventional disease-associated clonotypes will only be relevant in the carrier of the HLA allele to which the clonotype is restricted.

Beside their therapeutic utility, CAIT cells represent a promising platform to discover antigens implicated in the disease, for example, by focusing on antigens driving the expansion of CAIT cells. We previously established that CAIT cells respond to small molecules presented by CD1d including PPBF and CIPPBF [16], however, the nature of this presentation remains unclear. Specifically, whether this is a directed presentation of these small molecules by CD1d or does these molecules form a complex with other lipids and this complex is presented by CD1d molecules, that is, a hapten-like presentation. In addition, PPBF and CIPPBF are not naturally occurring molecules, hence, the exact metabolites or lipids driving the expansion of CAIT cells remain to be elucidated.

Here, we aimed not only to increase the sample size but also to include multiple cohorts spanning different stages of the disease. This not only enabled us to perform within-cohort analyses but also to perform a first-of-its-kind meta-analysis across these different cohorts. To this end, we were able to identify clonotypes that were specific to IBD in its early stage, late stage, and to the disease across all stages as revealed by the meta-analysis. One of the consistent signals across the different stages was the CAIT signal. This illustrates that these cells are a stable, robust marker of CD across the entire disease trajectory, particularly in individuals with ileal and ileocolonic CD. Further, it substantially highlights that the expansion of CAIT cells is not impacted by the different therapeutic trajectories and surgeries. Hence, deconvolving the antigenic specificity of these cells and discovering their roles in the disease is a promising strategy to understand the etiology of CD.

Large-scale TRB repertoire profiling studies across thousands of individuals have identified thousands of TRB clonotypes that are associated with the disease and simultaneously were able to discover their HLA restriction [35]. While our study represents the largest TRA analysis published to date, its sample size is smaller than these large TRB-based studies, and hence, its ability to identify disease-associated clonotypes is limited. One of the reasons that enabled us to identify CAIT cells with our relatively small sample size is their nature as unconventional T cells coupled with a large effect size. These T cells are not restricted by HLA proteins, which are highly polymorphic, but to the CD1d protein, which is monomorphic; hence, a relatively small sample size was able to associate them with the disease.

As large-scale bulk TCR repertoire profiling becomes a standard method to identify disease-associated clonotypes [20, 22, 36–38], the more urgent it becomes to identify the phenotypes, functions, and pathological roles of these clonotypes. Different research directions can be followed to discover and investigate these different aspects, such as the development of animal models to study the therapeutic potential of the targeted depletion of disease-associated clonotypes [7, 39, 40]. Given the semi-invariant TRA chain of CAIT cells, depleting these cells could be conducted by targeting their TRAV gene, specifically, the *TRAV12-1* gene. Although not all *TRAV12-1*^+^ are CAIT cells, all CAIT cells utilize this gene in their TRA chain. The therapeutic potential and side effects of such approaches need to be addressed first in animal studies. To improve the targeting of these cells, we envision the development of antibodies that target their TRA and TRB chains. Although the TRB chains of CAIT cells show a higher degree of diversity relative to their semi-invariant TRA chain, our previous investigation of paired TCR chains showed a degree of preferential usage of some TRBV genes [15]. Therefore, generating paired TCRs from CAIT cells would be a prerequisite step to improve the targeting and the characterization of these cells. Several methods can be used to generate the pairing, such as pairSEQ [41] and TIRTL-Seq [42] as well as single-cell RNA and TCR sequencing [15].

## Conclusions

Our analysis demonstrated that CAIT cells are significantly more expanded in individuals with CD, particularly in individuals who are ASCA^+^ and suffer from either ileal or ileocolonic CD and have severe disease complications. The expansion of these cells was not induced by the medications administered to control the disease, as they were significantly expanded in treatment-naive individuals. Neither did their expansion decrease with medications and surgery as they were highly expanded in individuals with CD, 20 years post-diagnosis. These findings highlight the importance of CAIT cells in CD and indicate that these cells might be relevant for understanding the etiopathology of CD as well as in developing therapies to treat and control the disease.

## Supplementary Information

Additional file 1. Supplementary Figures (Figs. S1-S4). Combined PDF containing all supplementary figures and corresponding legends.

Additional file 2. Supplementary Tables (Tables S1–S5). Excel file containing all supplementary tables, including phenotypic descriptions of the IBSEN-III, IBSEN-20, and BCBC cohorts, and lists of CD- and UC-associated TRA clonotypes.

## Data Availability

Due to GDPR and consent restrictions, the datasets reported in the current study, namely, the TCR-Seq datasets of the BCBC cohort, can be obtained by submitting a project application to the PopGen 2.0 Network (https://portal.popgen.de/). This dataset will be kept and made available for at least 10 years from the date of publication, and the processing time of applications is approximately 6-8 weeks. Regarding the IBSEN-III and the IBSEN-20 datasets, institutional data privacy regulations prohibit the deposition of individual-level data in public repositories. Participant written consent also does not cover public sharing of data for use for unknown purposes. Upon contact with Marte Lie Høivik (m.l.hoivik@medisin.uio.no), an institutional data transfer agreement can be established and data shared if the aims of data use are covered by ethical approval and patient consent. The procedure will involve an update to the ethical approval as well as a review by legal departments at both institutions, and the process will typically take one to two months from initial contact. The analytical code and software used in the current study are based on publicly available tools and custom scripts developed in Python, as described in the Methods section.

## References

[1] Jairath V, Feagan BG, Elsevier. Global burden of inflammatory bowel disease. Lancet Gastroenterol Hepatol. 2020;5:2–3. 10.1016/S2468-1253(19)30358-9. 31648974 10.1016/S2468-1253(19)30358-9

[2] Khan S, Sebastian SA, Parmar MP, Ghadge N, Padda I, Keshta AS, et al. Factors influencing the quality of life in inflammatory bowel disease: a comprehensive review. Dis-a-Mon. 2024;70:101672. 10.1016/j.disamonth.2023.101672. 10.1016/j.disamonth.2023.101672 38143196

[3] Marsal J, de Barreiro- Acosta M, Blumenstein I, Cappello M, Bazin T, Sebastian S. Management of non-response and loss of response to anti-tumor necrosis factor therapy in inflammatory bowel disease. Front Med. 2022. 10.3389/fmed.2022.897936. 10.3389/fmed.2022.897936 PMC9241563 35783628

[4] Alsoud D, Verstockt B, Fiocchi C, Vermeire S. Breaking the therapeutic ceiling in drug development in ulcerative colitis. Lancet Gastroenterol Hepatol. 2021;6:589–95. 10.1016/S2468-1253(21)00065-0. 34019798 10.1016/S2468-1253(21)00065-0

[5] Krickau T, Naumann-Bartsch N, Aigner M, Kharboutli S, Kretschmann S, Spoerl S, et al. CAR T-cell therapy rescues adolescent with rapidly progressive lupus nephritis from haemodialysis. Lancet. 2024;403:1627–30. 10.1016/S0140-6736(24)00424-0. 38642568 10.1016/S0140-6736(24)00424-0

[6] Ellebrecht CT, Bhoj VG, Nace A, Choi EJ, Mao X, Cho MJ, et al. Reengineering chimeric antigen receptor T cells for targeted therapy of autoimmune disease. Science. 2016;353:179–84. 10.1126/science.aaf6756. 27365313 10.1126/science.aaf6756 PMC5343513

[7] Britanova OV, Lupyr KR, Staroverov DB, Shagina IA, Aleksandrov AA, Ustyugov YY, et al. Targeted depletion of TRBV9+ T cells as immunotherapy in a patient with ankylosing spondylitis. Nat Med. 2023;29:2731–6. 10.1038/s41591-023-02613-z. 37872223 10.1038/s41591-023-02613-z PMC10667094

[8] ElAbd H, Pesesky M, Innocenti G, Chung BK, Mahdy AKH, Kriukova V, et al. T and B cell responses against Epstein–Barr virus in primary sclerosing cholangitis. Nat Med. 2025. 10.1038/s41591-025-03692-w. 40500415 10.1038/s41591-025-03692-w PMC12283410

[9] ElAbd H, Mahdy AKH. Decoding the etiology of immune-mediated inflammatory diseases statistically. Front Immunol. 2025. 10.3389/fimmu.2025.1610662. 40599790 10.3389/fimmu.2025.1610662 PMC12209366

[10] Goyette P, Boucher G, Mallon D, Ellinghaus E, Jostins L, Huang H, et al. High-density mapping of the MHC identifies a shared role for HLA-DRB1*01:03 in inflammatory bowel diseases and heterozygous advantage in ulcerative colitis. Nat Genet. 2015;47:172–9. 10.1038/ng.3176. 25559196 10.1038/ng.3176 PMC4310771

[11] Ahmad T, Armuzzi A, Bunce M, Mulcahy-Hawes K, Marshall SE, Orchard TR, et al. The molecular classification of the clinical manifestations of Crohn’s disease. Gastroenterology. 2002;122:854–66. 10.1053/gast.2002.32413. 11910336 10.1053/gast.2002.32413

[12] Degenhardt F, Mayr G, Wendorff M, Boucher G, Ellinghaus E, Ellinghaus D, et al. Transethnic analysis of the human leukocyte antigen region for ulcerative colitis reveals not only shared but also ethnicity-specific disease associations. Hum Mol Genet. 2021. 10.1093/hmg/ddab017. 33555323 10.1093/hmg/ddab017 PMC8098114

[13] Martini GR, Tikhonova E, Rosati E, DeCelie MB, Sievers LK, Tran F, et al. Selection of cross-reactive T cells by commensal and food-derived yeasts drives cytotoxic TH1 cell responses in Crohn’s disease. Nat Med. 2023;29:2602–14. 10.1038/s41591-023-02556-5. 37749331 10.1038/s41591-023-02556-5 PMC10579100

[14] Uchida AM, Boden EK, James EA, Shows DM, Konecny AJ, Lord JD. *Escherichia coli*–specific CD4+ T cells have public T-cell receptors and low interleukin 10 production in Crohn’s disease. Cell Mol Gastroenterol Hepatol. 2020;10:507–26. 10.1016/j.jcmgh.2020.04.013. 32361018 10.1016/j.jcmgh.2020.04.013 PMC7385044

[15] Rosati E, Martini GR, Pogorelyy MV, Minervina AA, Degenhardt F, Wendorff M, et al. A novel unconventional T cell population enriched in Crohn’s disease. Gut. 2022;71:2194–204. 10.1136/gutjnl-2021-325373. 35264446 10.1136/gutjnl-2021-325373 PMC9554086

[16] Minervina AA, Pogorelyy MV, Paysen S, Luening U, Degenhardt F, Franke A, et al. Crohn’s-associated invariant T cells (CAITs) recognise small sulfonate molecules on CD1d. Gut. 2022. 10.1136/gutjnl-2022-328684. 36428091 10.1136/gutjnl-2022-328684 PMC10715465

[17] Allez M, Auzolle C, Ngollo M, Bottois H, Chardiny V, Corraliza AM, et al. T cell clonal expansions in ileal Crohn’s disease are associated with smoking behaviour and postoperative recurrence. Gut. 2019;68:1961–70. 10.1136/gutjnl-2018-317878. 30792246 10.1136/gutjnl-2018-317878

[18] Doorenspleet ME, Westera L, Peters CP, Hakvoort TBM, Esveldt RE, Vogels E, et al. Profoundly expanded T-cell clones in the inflamed and uninflamed intestine of patients with Crohn’s disease. J Crohns Colitis. 2017;11:831–9. 10.1093/ecco-jcc/jjx012. 28158397 10.1093/ecco-jcc/jjx012

[19] Mahdy AKH, Lokes E, Schöpfel V, Kriukova V, Britanova OV, Steiert TA, et al. Bulk T cell repertoire sequencing (TCR-Seq) is a powerful technology for understanding inflammation-mediated diseases. J Autoimmun. 2024;149:103337. 10.1016/j.jaut.2024.103337. 39571301 10.1016/j.jaut.2024.103337

[20] Emerson RO, DeWitt WS, Vignali M, Gravley J, Hu JK, Osborne EJ, et al. Immunosequencing identifies signatures of cytomegalovirus exposure history and HLA-mediated effects on the T cell repertoire. Nat Genet. 2017;49:659–65. 10.1038/ng.3822. 28369038 10.1038/ng.3822

[21] Gittelman RM, Lavezzo E, Snyder TM, Zahid HJ, Carty CL, Elyanow R, et al. Longitudinal analysis of T cell receptor repertoires reveals shared patterns of antigen-specific response to SARS-CoV-2 infection. JCI Insight. 2022. 10.1172/jci.insight.151849. 35439174 10.1172/jci.insight.151849 PMC9220833

[22] Greissl J, Pesesky M, Dalai SC, Rebman AW, Soloski MJ, Horn EJ, et al. Immunosequencing of the T-cell receptor repertoire reveals signatures specific for identification and characterization of early lyme disease. medRxiv. 2022;2021.07.30.21261353. 10.1101/2021.07.30.21261353.

[23] Kristensen VA, Opheim R, Perminow G, Huppertz-Hauss G, Detlie TE, Lund C, et al. Inflammatory bowel disease in South-Eastern Norway III (IBSEN III): a new population-based inception cohort study from South-Eastern Norway. Scand J Gastroenterol. 2021;56:899–905. 10.1080/00365521.2021.1922746. 34154494 10.1080/00365521.2021.1922746

[24] Solberg IC, Lygren I, Jahnsen J, Aadland E, Høie O, Cvancarova M, et al. Clinical course during the first 10 years of ulcerative colitis: results from a population-based inception cohort (IBSEN study). Scand J Gastroenterol. 2009;44:431–40 Taylor & Francis. 10.1080/00365520802600961. 19101844 10.1080/00365520802600961

[25] Bolotin DA, Poslavsky S, Mitrophanov I, Shugay M, Mamedov IZ, Putintseva EV, et al. MiXCR: software for comprehensive adaptive immunity profiling. Nat Methods. 2015;12:380–1. 10.1038/nmeth.3364. 25924071 10.1038/nmeth.3364

[26] Whitlock MC. Combining probability from independent tests: the weighted Z-method is superior to Fisher’s approach. J Evol Biol. 2005;18:1368–73. 16135132 10.1111/j.1420-9101.2005.00917.x

[27] Fisher RA. Statistical Methods for Research Workers. In: Kotz S, Johnson NL, editors. Breakthroughs in Statistics: Methodology and Distribution. New York, NY: Springer New York; 1992. p. 66–70. 10.1007/978-1-4612-4380-9_6.

[28] Shannon P, Markiel A, Ozier O, Baliga NS, Wang JT, Ramage D, et al. Cytoscape: a software environment for integrated models of biomolecular interaction networks. Genome Res. 2003;13:2498–504. 10.1101/gr.1239303. 14597658 10.1101/gr.1239303 PMC403769

[29] Edwards AWF. RA Fischer, statistical methods for research workers, (1925). Landmark writings in western mathematics 1640-1940. Elsevier; 2005. p. 856–70. 10.1007/978-1-4612-4380-9_6.

[30] Mahdy AKH, Schöpfel V, Huppertz-Hauss G, Perminow G, Tran F, Bang C, et al. Simultaneous profiling of the blood and gut T and B cell repertoires in Crohn’s disease and symptomatic controls illustrates tissue-specific alterations in the immune repertoire of individuals with Crohn’s disease. Front Immunol. 2025. 10.3389/fimmu.2025.1638522. 40977727 10.3389/fimmu.2025.1638522 PMC12447526

[31] Wei M, Wu J, Bai S, Zhou Y, Chen Y, Zhang X, et al. TRAIT: A Comprehensive Database for T-cell Receptor-Antigen Interactions. bioRxiv. 2024;2024.11.20.624436. 10.1101/2024.11.20.624436 10.1093/gpbjnl/qzaf033 PMC12448929 40257421

[32] Bourgonje AR, Andreu-Sánchez S, Vogl T, Hu S, Vich Vila A, Gacesa R, et al. Phage-display immunoprecipitation sequencing of the antibody epitope repertoire in inflammatory bowel disease reveals distinct antibody signatures. Immunity. 2023;56:1393-1409.e6. 10.1016/j.immuni.2023.04.017. 37164015 10.1016/j.immuni.2023.04.017

[33] Faye AS, Allin KH, Iversen AT, Agrawal M, Faith J, Colombel J-F, et al. Antibiotic use as a risk factor for inflammatory bowel disease across the ages: a population-based cohort study. Gut. 2023;72:663. 10.1136/gutjnl-2022-327845. 36623926 10.1136/gutjnl-2022-327845 PMC9998355

[34] Ebert AC, Harper S, Vestergaard MV, Mitchell W, Jess T, Elmahdi R. Risk of inflammatory bowel disease following hospitalisation with infectious mononucleosis: nationwide cohort study from Denmark. Nat Commun. 2024;15:8383. 10.1038/s41467-024-52195-8. 39333475 10.1038/s41467-024-52195-8 PMC11437054

[35] Pesesky M, Bharanikumar R, Le Bourhis L, ElAbd H, Rosati E, Carty CL, et al. Antigen-driven expansion of public clonal T cell populations in inflammatory bowel diseases. J Crohns Colitis. 2025;jjaf048. 10.1093/ecco-jcc/jjaf048. 40121186 10.1093/ecco-jcc/jjaf048

[36] Nolan S, Vignali M, Klinger M, Dines JN, Kaplan IM, Svejnoha E, et al. A large-scale database of T-cell receptor beta (TCRβ) sequences and binding associations from natural and synthetic exposure to SARS-CoV-2. Res Sq. American Journal Experts; 2020. 10.3389/fimmu.2025.1488851. 10.3389/fimmu.2025.1488851 PMC11873104 40034696

[37] Faham M, Carlton V, Moorhead M, Zheng J, Klinger M, Pepin F, et al. Discovery of T cell receptor β motifs specific to HLA–B27–positive ankylosing spondylitis by deep repertoire sequence analysis. Arthritis Rheum. 2017;69:774–84. 10.1002/art.40028. 10.1002/art.40028 28002888

[38] Rawat P, Shapiro MR, Peters LD, Widrich M, Mayer-Blackwell K, Motwani K, et al. Identification of a type 1 diabetes-associated T cell receptor repertoire signature from the human peripheral blood. medRxiv. 2024;2024.12.10.24318751. 10.1101/2024.12.10.24318751. 10.1126/sciadv.adx7448 PMC12904210 41686902

[39] Chiocchia G, Boissier M-C, Fournier C, John Wiley & Sons, Ltd. Therapy against murine collagen-induced arthritis with T cell receptor Vβ-specific antibodies. Eur J Immunol. 1991;21:2899–905. 10.1002/eji.1830211202. 1836185 10.1002/eji.1830211202

[40] Liu Z, Cort L, Eberwine R, Herrmann T, Leif JH, Greiner DL, et al. Prevention of type 1 diabetes in the rat with an allele-specific anti–T-cell receptor antibody: Vβ13 as a therapeutic target and biomarker. Diabetes. 2012;61:1160–8. 10.2337/db11-0867. 22368175 10.2337/db11-0867 PMC3331757

[41] Howie B, Sherwood AM, Berkebile AD, Berka J, Emerson RO, Williamson DW, et al. High-throughput pairing of T cell receptor α and β sequences. Sci Transl Med. 2015;7:301ra131-301ra131. 10.1126/scitranslmed.aac5624. 10.1126/scitranslmed.aac5624 26290413

[42] Pogorelyy MV, Kirk AM, Adhikari S, Minervina AA, Sundararaman B, Vegesana K, et al. TIRTL-seq: Deep, quantitative, and affordable paired TCR repertoire sequencing. bioRxiv. 2024:2024.09.16.613345. 10.1101/2024.09.16.613345. 10.1038/s41592-025-02907-9 PMC12791010 41286199

