# Supplementary material for: Multi-centered T cell repertoire profiling identifies alterations in the immune repertoire of individuals with inflammatory bowel disease across different disease stages
Source: Genome Med. 2026 Jan 9;18:3. doi: 10.1186/s13073-025-01575-w (PMC12784487; doi:10.1186/s13073-025-01575-w)
Supplement: Supplementary file 1 — Additional file 1. Supplementary Figures (Figs. S1-S4). Combined PDF containing all supplementary figures and corresponding legends. [file 13073_2025_1575_MOESM1_ESM.pdf]

Supplementary figures

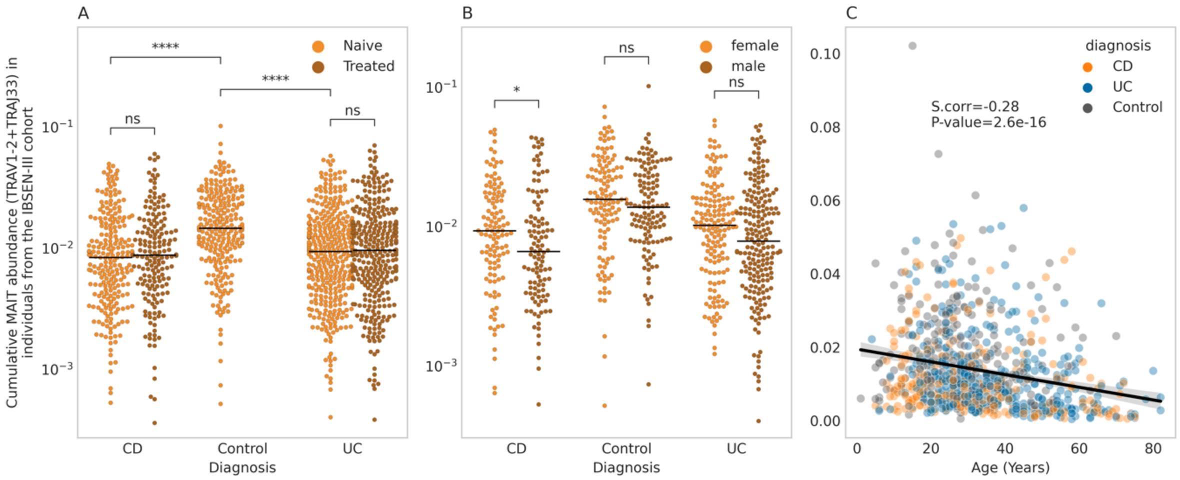

Figure S1: *The impact of IBD, therapy, biological sex, and age on the expansion of MAIT cells*. (**A**) the expansion of MAIT cells in individuals from the IBSEN-III cohort, namely, treatment-naive and treated individuals with CD and UC, and symptomatic controls. (**B**) shows the impact of biological sex on the expansion of MAIT cells in treatment-naive individuals, while (**C**) shows the strong negative correlation between the expansion of MAIT cells and age in years, regardless of disease status.

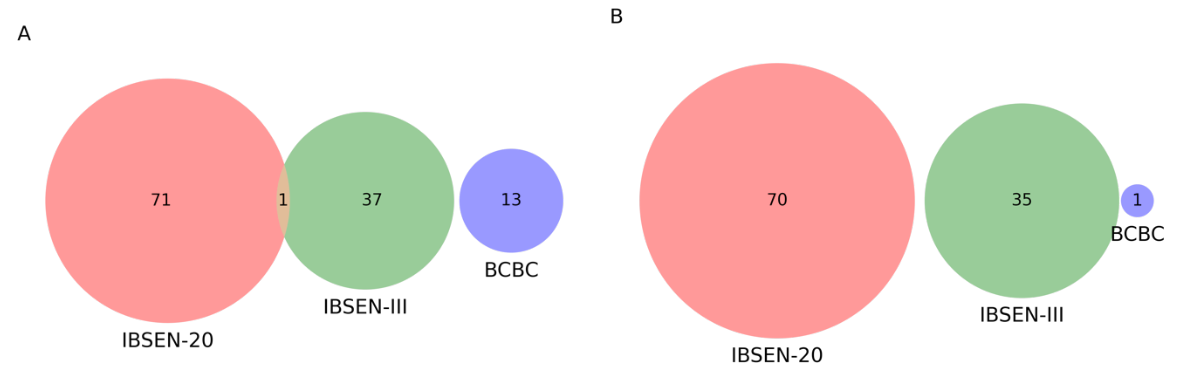

Figure S2: *The overlap among disease-associated clonotypes identified by analyzing each cohort independently.* (**A**) depicts the overlap among CD-associated clonotypes, while (**B**) depicts the overlap among UC-associated clonotypes.

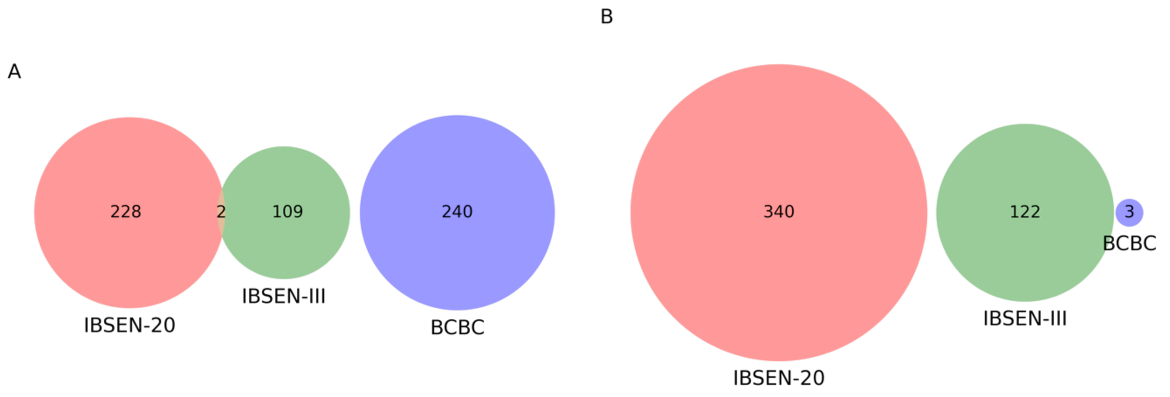

Figure S3: *The overlap in CD- and UC- associated meta-clonotypes identified using seeded-clustering.* (**A**) illustrate the overlap at the level of CD-associated meta-clonotypes, while (**B**) shows the overlap among the UC-associated meta-clonotypes.

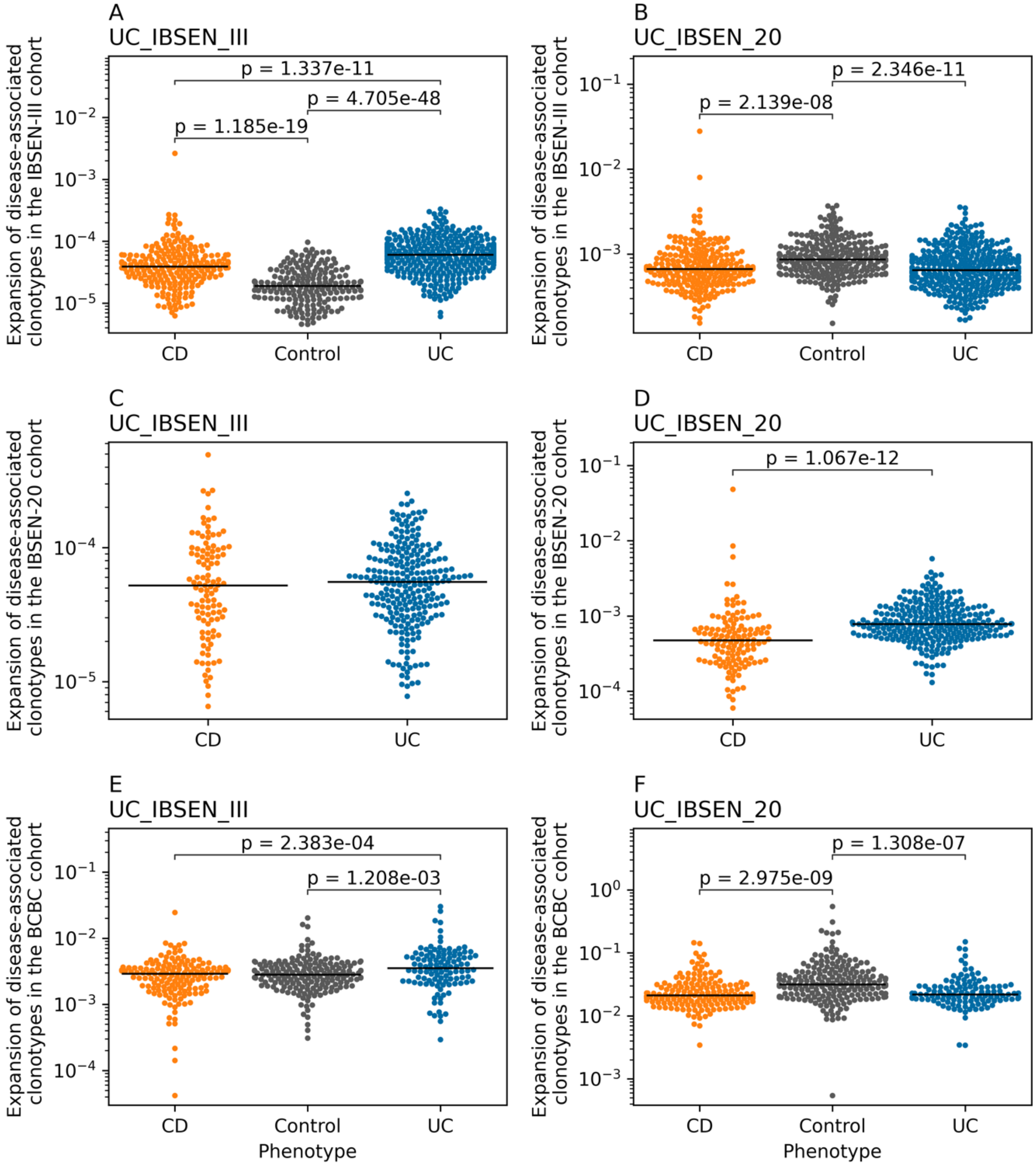

Figure S4*: The expansion of the different UC-associated clonotypes in the three discovery cohorts, namely, IBSEN-III, IBSEN-20, and BCBC.* (**A**) and (**B**) show the expansion of the UC-associated clonotype sets identified from the analysis of the IBSEN-III cohort (UC_IBSEN_III) and IBSEN-20 cohort (UC_IBSEN_20) in the TRA repertoire of IBSEN-III. (**C**) and (**D**) show the expansion of the identified UC-associated clonotype sets in the repertoire of IBSEN-20 individuals. Lastly, (**E**) and (**F**) depict the expansion of UC-associated clonotype sets across individuals included in the BCBC cohort as well as in the included population controls. Across all panels, statistical comparisons were conducted using the two-sided Mann-Whitney U test.

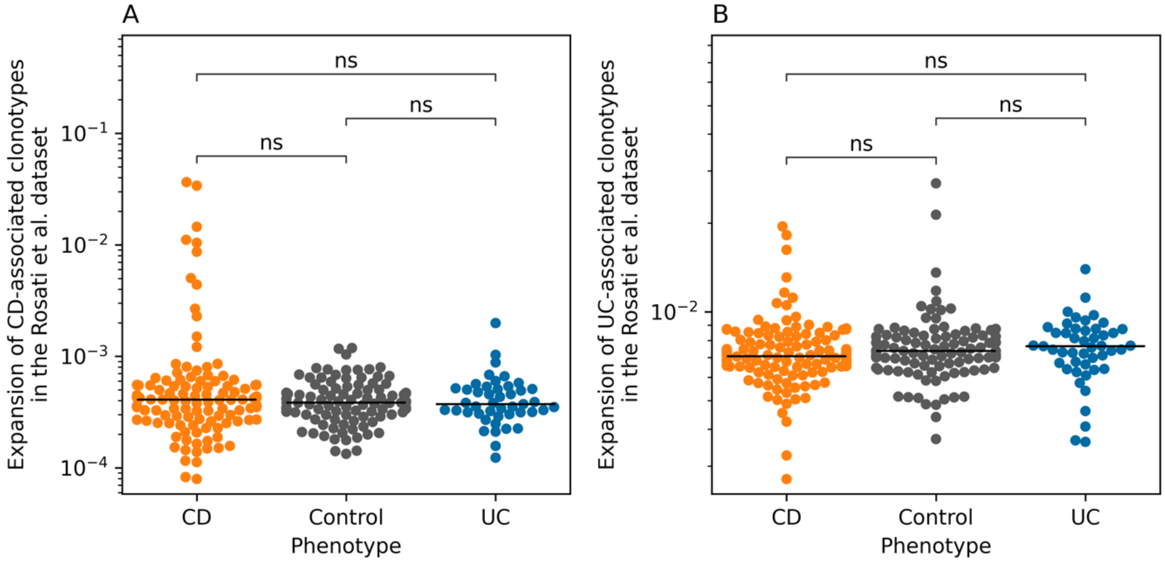

Figure S5: *The expansion of the CD- and UC- associated clonotypes identified by integrating the TRA of the three cohorts in the Rosati et al.* [1] *datasets*. (**A**) shows the expansion of CD-associated clonotypes, while (**B**) shows the expansion of UC-associated clonotypes.

References

1. Rosati E, Martini GR, Pogorelyy M V, Minervina AA, Degenhardt F, Wendorff M, et al. A novel unconventional T cell population enriched in Crohn’s disease. Gut [Internet]. 2022;71:2194 LP – 2204. https://doi.org/10.1136/gutjnl-2021-325373
